# Graphene-Induced Pore Formation on Cell Membranes

**DOI:** 10.1038/srep42767

**Published:** 2017-02-20

**Authors:** Guangxin Duan, Yuanzhao Zhang, Binquan Luan, Jeffrey K. Weber, Royce W. Zhou, Zaixing Yang, Lin Zhao, Jiaying Xu, Judong Luo, Ruhong Zhou

**Affiliations:** 1 Institute of Quantitative Biology and Medicine, SRMP and RAD-X, Collaborative Innovation Center of Radiation Medicine of Jiangsu Higher Education Institutions, Soochow University, Suzhou 215123, China; 2 IBM Thomas J. Watson Research Center, Yorktown Heights, NY 10598, USA; 3 Department of Oncology, The Affiliated Hospital of Nanjing Medical University, Changzhou No.2 People’s Hospital, Changzhou, 213003, China; 4 Department of Chemistry, Columbia University, New York, NY 10027, USA

## Abstract

Examining interactions between nanomaterials and cell membranes can expose underlying mechanisms of nanomaterial cytotoxicity and guide the design of safer nanomedical technologies. Recently, graphene has been shown to exhibit potential toxicity to cells; however, the molecular processes driving its lethal properties have yet to be fully characterized. We here demonstrate that graphene nanosheets (both pristine and oxidized) can produce holes (pores) in the membranes of A549 and Raw264.7 cells, substantially reducing cell viability. Electron micrographs offer clear evidence of pores created on cell membranes. Our molecular dynamics simulations reveal that multiple graphene nanosheets can cooperate to extract large numbers of phospholipids from the membrane bilayer. Strong dispersion interactions between graphene and lipid-tail carbons result in greatly depleted lipid density within confined regions of the membrane, ultimately leading to the formation of water-permeable pores. This cooperative lipid extraction mechanism for membrane perforation represents another distinct process that contributes to the molecular basis of graphene cytotoxicity.

Graphene’s remarkable physicochemical properties have long garnered favor among scientists seeking stable, electrically conductive, and optically active 2D nanomaterials. Numerous studies have demonstrated wide-reaching prospects for biomedical applications of graphene and graphene oxide (GO), particularly in biosensing^1^,^2^, tumor imaging^3^,^4^,^5^, drug and gene delivery^6^,^7^,^8^,^9^, tumor photothermal therapy^10^,^11^,^12^ and bactericidal agency^13^,^14^.

The introduction of graphene-based nanomaterials into human-proximate systems has prompted efforts to understand graphene’s biocompatibility and cytotoxicity. Much of the existing literature attributes GO’s cytotoxicity to a secondary generation of reactive oxygen species (ROS)^15^,^16^,^17^: GO has been shown to elicit oxidative stress in cells, even at low concentrations, and in a time- and concentration-dependent manner. However, recent work also indicates that GO can directly damage cells via interactions with various biomacromolecules^18^,^19^,^20^,^21^,^22^,^23^,^24^. Matesanz and co-workers discovered that GO can localize on F-actin filaments after cellular uptake, inducing cell cycle arrest and apoptosis^21^. GO nanosheets were also found to interact with electron transport chain complexes^23^, reducing ATP synthesis and inhibiting cellular migration and activity. Previous *in-vitro* study of GO’s interaction with a lipid vesicle suggested potential damage of cell membrane^25^. Our previous study featuring both molecular dynamics (MD) simulations and transmission electron microscopy (TEM) revealed that, in addition to penetrating cell membranes, GO can directly extract phospholipid molecules from membrane bilayers^18^,^26^. Another study indicated that both pristine graphene and GO can disrupt protein-protein interactions by splitting protein-protein dimers^20^. Zhang and coworkers recently reported observations of enhanced membrane permeability after the insertion of micrometer-sized graphene oxides (mGOs) into cell membranes; they also noted vacuole formation resulting from interactions between mGOs and membrane-embedded aquaporins^27^. Furthermore, Qu *et al*. found that GO could interact with Toll-like receptor 4 (TLR-4) and induce necrosis in macrophages by increasing the expression of TNF-α^22^.

Accumulating experimental and computational evidence thus suggests that GO nanotoxicity is driven by multiple molecular processes. In that light, coarse-grained, mean-field simulations have also suggested the possibility of graphene-mediated perforation of cell membranes, a phenomenon likely to be cytotoxic^28^. Here, we report direct observations of such GO-induced pore formation on cell membranes as imaged with optical, fluorescence, and scanning electron microscopy (SEM) and supported by molecular dynamics (MD) simulations. Our MD results highlight a striking mechanism by which multiple graphene nanosheets cooperate to extract lipids and create pores in interstitial regions of dense graphene assemblies.

## Results and Discussion

### Characterization of GO

The morphologies of the GO nanosheets used in this study were first examined by atomic force microscopy (AFM). AFM images revealed a characteristic GO thickness of around 1 nm (Figure S1), implying a single-layered GO architecture consistent with those seen in previous studies^29^,^30^. The lateral sizes of the GO sheets were observed to range from 200 nm to 700 nm. UV and Raman spectroscopy were employed to probe electronic and vibrational nanosheet characteristics. As shown in Figure S2, a dominant UV absorbance peak appeared at ~230 nm, a wavelength consistent with past results^31^,^32^. Raman spectra exhibited characteristic D and G bands at ~1350 and 1598 cm^−1^, respectively^31^,^33^. Considered together, these data indicate that the GO solutions used in our experiments were mostly populated by single-layered nanosheets.

### Cytotoxicity of GO to both A549 and Raw264.7 cells

**Figure 1:**
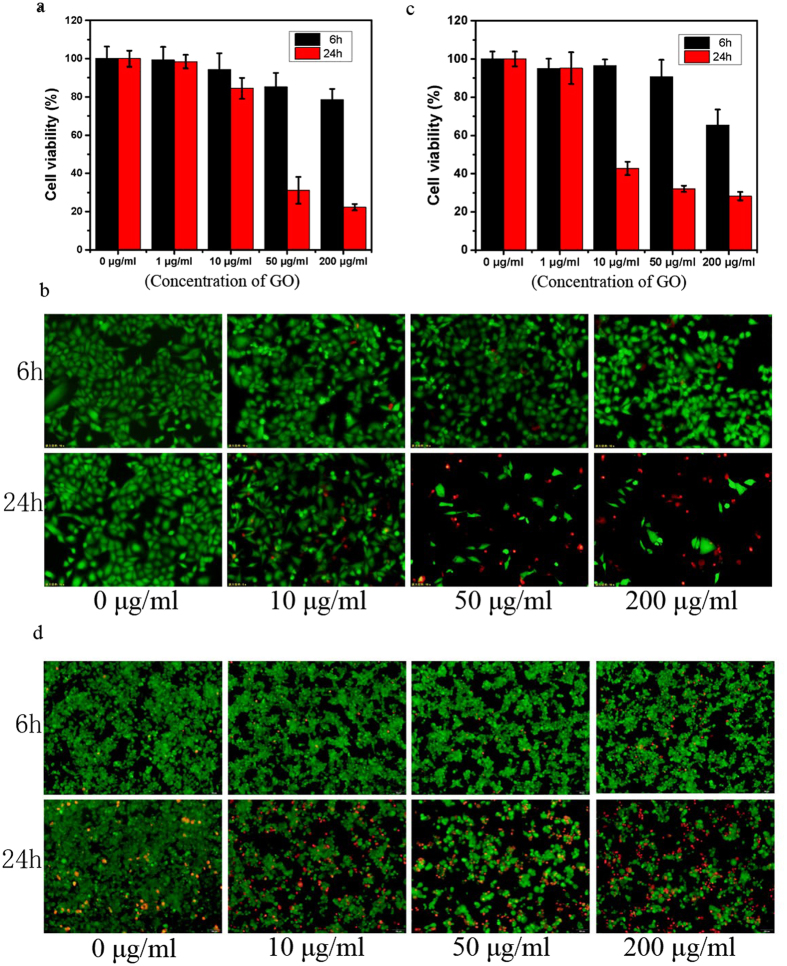
Toxicity of GO to both A549 and Raw264.7 cells subjected to various GO concentrations (ranging from 1 μg/ml to 200 μg/ml) and incubation times (6 h or 24 h). (**a**) CCK-8 assay for A549; (**b**) live/dead assay for A549; (**c**) CCK-8 assay for Raw264.7; (**d**) live/dead assay for Raw264.7.

In previous work, we demonstrated that complete culture medium containing serum proteins can mitigate the cytotoxicity of GO^26^,^30^. We here, however, focus on the cytotoxicity of GO in a serum protein-free environment. In order to evaluate the cytotoxicity of GO to mammalian cells, we chose to study human lung A549 cells and murine Raw264.7 macrophages, which are widely used in nanotoxicity experiments^15^,^34^,^35^,^36^,^37^,^38^. The A549 and Raw264.7 cells were first incubated in complete culture medium containing 10% fetal bovine serum (FBS). After a 24 hour incubation period, both cell lines reached ~80% confluence; at that point, the cells were exposed to GO nanosheets for either 6 or 24 hours in serum-free medium (0% FBS). The CCK-8 cell survival assay was the primary tool used to assess GO cytotoxicity. Figure 1 illustrates the toxic effects of GO on the two cell lines: overall, cell viabilities displayed negative time and GO-concentration dependence. Both A549 and Raw264.7 cells exhibited very low viabilities after 24 hours of incubation at relatively high GO concentrations (50 to 200 μg/ml – Fig. 1a and c), an observation well-aligned with our previous results^26^. To confirm the results of our CCK-8 assays, we also performed live/dead assays on both cell lines following GO treatment (Fig. 1b and d). These live/dead measurements yielded results consistent with our CCK-8 data: over 24 hours of incubation with 50 to 200 μg/ml GO, the majority of A549 and Raw264.7 cells were determined to have been killed (Fig. 1b and d). Our results thus indicate that GO is quite toxic to both of the mammalian cell lines studied.

### Cell surface morphologies indicate pore formation after GO treatment

**Figure 2:**
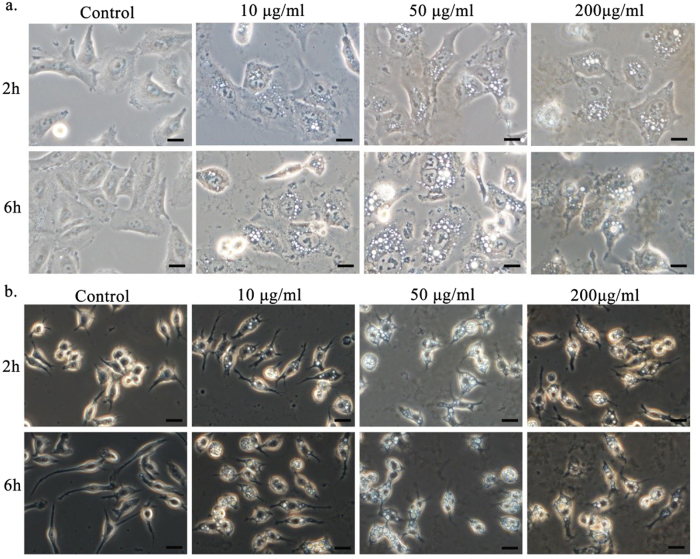
Morphologies of A549 (**a**) and Raw264.7 (**b**) cells as observed by optical microscopy after GO treatment, across different incubation times and GO concentrations (scale bar = 20 μm).

In order to explore mechanisms of GO-related cytotoxicity, cellular morphology was first measured after GO nanosheet exposure. Interestingly, light spots were detected in the membranes of both A549 (Fig. 2a) and Raw264.7 (Fig. 2b) cells subjected to GO treatment. Under an inverted microscope, light spots (or pores/holes, see below) could be identified after only one hour in 10 μg/ml GO solutions, which became clearly visible after two hours (Figs 2 and S3). Light spots continued to expand in both size and number as the incubation time increased, and exceptionally dense spots appeared at the highest GO concentrations (Fig. 2, S3, S4 and Movie S1). As shown in Figure S4, the average number of light spots on each A549 cell was approximately 23.6 ± 6.6, 44.1 ± 8.7, 51.7 ± 9.9, respectively, after 2 h incubation with 10, 50, and 200 μg/ml GO, and then it grew to 35.0 ± 8.1, 68.5 ± 8.6, 32.5 ± 10.9, respectively, after 6 h. It is striking to note that up to ~70 light spots (with sizes from tens to hundreds of nm) per A549 cell could be detected after 6 h incubation with 50 μg/ml GO. However, the number of light spots decreased significantly after treated with a much higher concentration of 200 μg/ml GO for 6 h, which may attribute to shrinkage and death of cells (Fig. 2, S3, S4). It was demonstrated in Movie S1 that recorded by the live-cell imaging system, light spots emerged after GO-treating for 2 h and became larger and larger in the rest of incubation time. Then, because of the significant injury caused by GO, cells went to death. Thus, deformation of cells via possible membrane disruption by GO was detected. Similar light spots were found in other GO-treated cell lines, such as Beas-2b, HUVEC, and HepG2 cells, which demonstrated that light spots induced by GO should be a common phenomenon; however, these spots were more prominent on A549 and Raw 264.7 cells (Figure S5).

Further analysis showed that GO-induced light spots appear even in the presence of serum protein medium (10% FBS), albeit after longer treatment times and at higher GO concentrations (Figure S6). However, it is interesting to note that treatment with PEGylated GO (PEG-GO; see Figure S7 for its characterization) did not result in light spot formation, even after A549 cells were treated with 200 μg/ml PEG-GO for 24 hours. A CCK-8 assay also confirmed that PEG-GO had no noticeable cytotoxicity (Figure S8), an observation consistent with our recent experimental and simulation studies that demonstrated PEG-GO inflicts very limited damage on macrophage cell membranes^39^.

**Figure 3:**
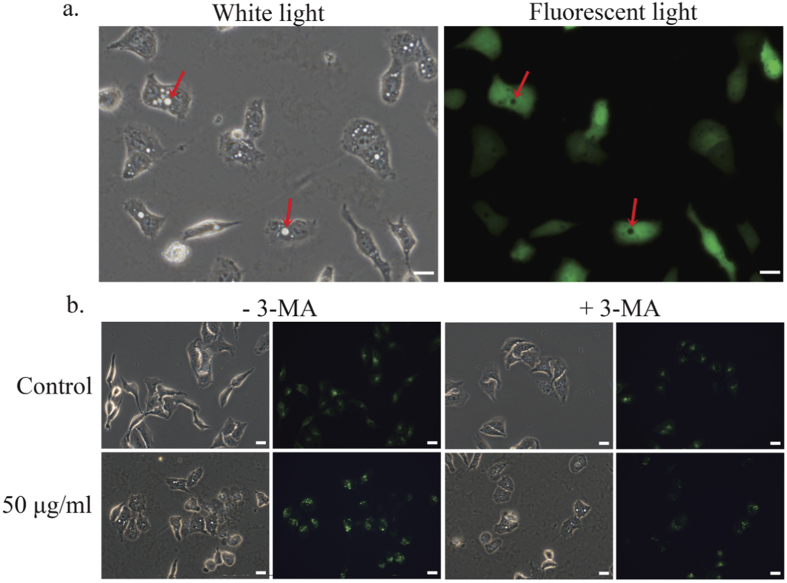
Optical and fluorescence micrographs of cellular damage induced by GO. (**a**) A549*-*GFP detected by optical and fluorescence microscopy after GO treatment (50 μg/ml; 6 h). (**b**) Autophagy detected by optical and fluorescence microscopy in A549-GFP cells; 3-MA, an autophagosome inhibitor, was added after GO treatment for 6 h. Scale bar = 20 μm.

To better understand the origin of the light spots seen with inverted light microscopy, A549 cells transfected with green fluorescent protein (GFP) were probed with fluorescence microscopy. The light spots clearly correspond to dark regions under fluorescence-based imaging (Fig. 3a), suggesting that these features represent holes (pores) in cell membranes. One possibility with these putative holes is that the observed features could be related to autophagy. To evaluate whether autophagy was involved, we performed a monodansylcadaverine (MDC) staining experiment on cells exposed to GO. After 6 hours of incubation in 50 μg/ml GO, we detected increased numbers of both autophagosomes and light spots; however, the two did not colocalize. We further introduced 3-MA, a common inhibitor of autophagy, into GO-incubated cell solutions, which significantly decreased the presence of autophagosomes; however, the light spots were unaffected by this addition (Fig. 3b). This evidence suggests that the observed light spots are neither autophagosomes themselves nor directly caused by autophagy.

### SEM confirms perforation after GO exposure

**Figure 4:**
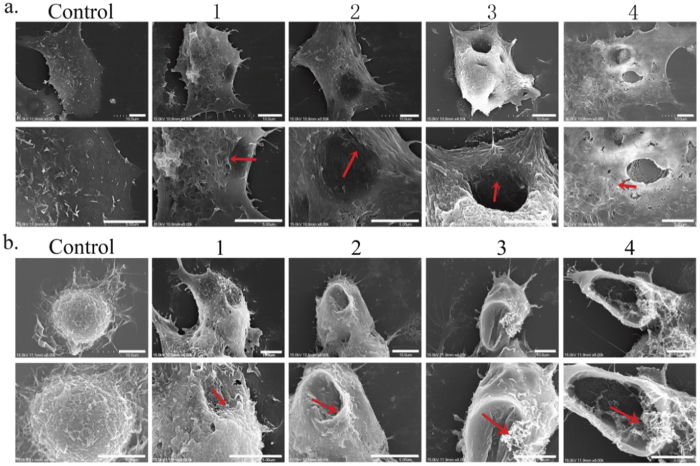
SEM images of cell membrane damage (in its final stage, >24 h) incurred by A549 (**a**) and Raw264.7 (**b**) cells as a result of GO exposure. The subfigure indices 1, 2, 3 and 4 represent progressive degrees of membrane stress observed during different phases of incubation. Scale bar = 5 μm.

After excluding a relationship to autophagy, we hypothesized that the formation of light spots is related to cell membrane stress induced by GO. In order to validate this premise, GO-treated cell membranes were imaged by scanning electron microscopy (SEM). In a previous study by Zhang and coworkers^27^, TEM imaging demonstrated that micrometer-sized GO sheets (mGO) can induce the formation of vacuoles in the cytosol and enhance cell membrane permeability. However, direct membrane perforation was not observed. In contrast to these TEM results^27^, we obtained scanning electron micrographs (SEM) that provide conclusive evidence of pore formation on A549 and Raw264.7 cell membranes. SEM images clearly show that many pores of various sizes (ranging from tens of nanometers to a few micrometers that can be affected by graphene sizes and membrane proteins) can be observed on cell membranes once exposed to GO (with those most obvious ones displayed in Fig. 4; some small pores can also merge into a larger one). Remarkably, GO nanosheets (marked by arrows) could also be detected inside or around the membrane pores in these SEM images. The progression of pore formation can also be roughly inferred from micrographs obtained at the different stages of GO incubation (Fig. 4a and b). Although no perforation of cell membranes was seen in our previous TEM images^18^ (likely due to their limited resolution), similarly staged damage — occurring in roughly three phases — was also observed in those experiments^18^. In both cases, these gradated processes appear to inflict significant damage on cell membranes and ultimately lead to cell death.

### MD simulations reveal an underlying molecular mechanism for perforation

As mentioned above, our previous work revealed that strong dispersion interactions between lipids and graphene can result in an extraordinary extraction of phospholipids from cell membranes, providing a dominant mechanism for the death of cells bygraphene nanosheets^18^,^40^. However, in previous simulations involving single graphene sheets, no pore formation in the lipid bilayer was observed. The possibility of pore formation has been suggested by past experiments, and membrane perforation has been posited to play an important role^28^. The current study demonstrates obvious pore formation on both live (Figs 2 and 4), and dead (Figure S9) cells.

This apparent discrepancy between past simulation results and our present observations of membrane perforation can perhaps be understood in terms of graphene nanosheet density. In our previous simulations, the effective graphene density was very low (projected across periodic boundary conditions), meaning extracted lipids could easily be replenished by neighboring molecules. *In vitro*, and particularly at high graphene concentrations, multiple graphene nanosheets could certainly interact with a local membrane segment simultaneously. In principle, these cooperative interactions could result in a critical loss of membrane density, leading to perforations at lipid-depleted sites. Here, we indeed introduce a simple (small yet proof-of-principle) system wherein multiple graphene molecules cooperate to weaken a membrane and induce pore formation at a nanosheet-interstitial site.

Atomistic MD simulations, widely used to characterize interactions between and among both biomolecules^41^,^42^,^43^,^44^,^45^ and nanomaterials^46^,^47^,^48^,^49^,^50^, proved useful for studying the GO-induced membrane perforation investigated here. It should be noted that, to simplify our models, we used pristine graphene nanosheets in our MD simulations. GO is favored in experiments primarily due to the poor water solubility of pristine graphene. In our previous study on cell membrane/graphene interactions^18^, we also simulated GO nanosheets according to the Lerf-Klinowski model (C_10_O_1_(OH)_1_(COOH)_0.5_; i.e., 2 epoxy and 2 hydroxyl groups on both sides of the graphene basal plane and 1 carboxyl group on the edge of the graphene sheet per 20 carbon atoms, yielding a C:O ratio about 3:1), which represents a typical outcome of graphene oxidation reactions^51^,^52^,^53^. Our results related to GO nanosheets were largely consistent with those derived from pristine graphene, as GO exhibited only slightly less pronounced interactions with cell membranes^18^. It is also noteworthy that large unoxidized regions can persist on GO nanosheets (so-called “*sp2*-domains”)^51^,^54^,^55^, with up to ~60% of the surface remaining undisturbed and graphene-like^54^. The pristine graphene used in our simulations thus mimics the *sp2*-domains of GOs.

**Figure 5:**
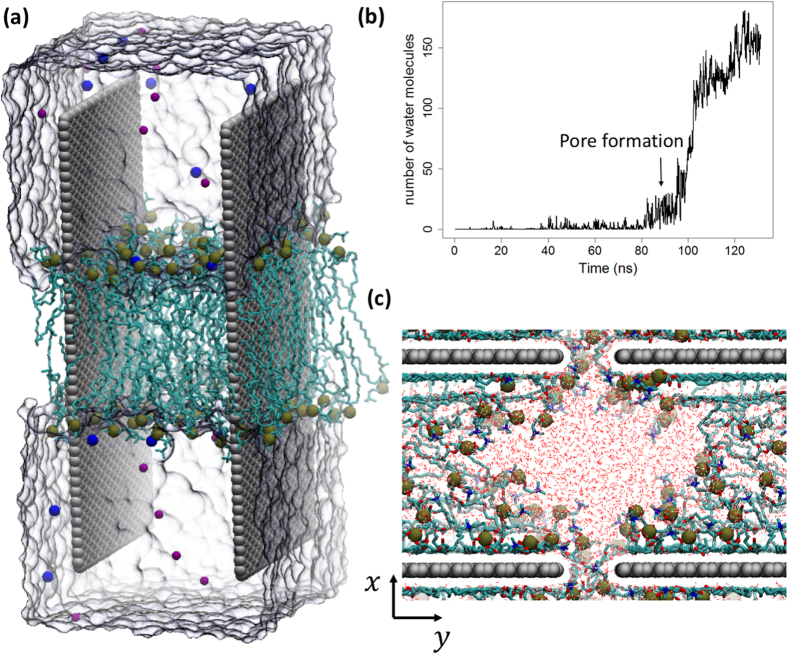
(**a**) Side view of the initial configuration of the simulation system. Two graphene nanosheets (rendered in gray vdW spheres) were inserted into a lipid membrane in a parallel orientation and at a separation of 4 nm. The system was then solvated in a 15 nm × 6 nm × 6 nm water box, which is shown using a surface representation. (**b**) Number of water molecules that have penetrated into the membrane (defined as those molecules that have come within 3 Å of the geometric center of the membrane in the vertical direction) as a function of time. Before the membrane is punctured, this number stays close to zero; after the perforation, the influx of water increases dramatically as the pore expands. (**c**) Top view of the membrane-perforated system after 120 ns of MD simulation. Water molecules (shown in a line representation) clearly occupy the inside of the membrane pore. The system has been shifted by a half-image length in the horizontal direction so that the pore can be easily recognized.

Due to the significant computational resources required for atomistic simulations, we featured a much smaller graphene sheet (~10 nm) in our simulations than we used in our experiments (200–700 nm). As demonstrated by the data shown in Figure S10, however, both the number and extent of membrane pores increases with increasing GO size. We thus feel assured in speculating that, if membrane perforation can be observed in a small simulation system, such pore formation processes could be even more prominent in larger systems. Contained within a 15 nm × 6 nm × 6 nm water box, our simulation system includes two parallel graphene nanosheets (10.5 nm × 4.5 nm) inserted vertically into a phospholipid bilayer (Fig. 5a). The two sheets were configured face-on at a 4 nm separation and restrained. Notably, enforcement of periodic boundary conditions yields an effective quartet of graphene sheets in complex, as rendered in Fig. 5. After equilibrating the system, we performed three independent production simulations reaching an aggregate length of 0.5 μs. Strikingly, pore formation (accompanied by water permeation through the membrane) was observed in all three trajectories (Fig. 5b and c; see also Figure S11).

**Figure 6:**
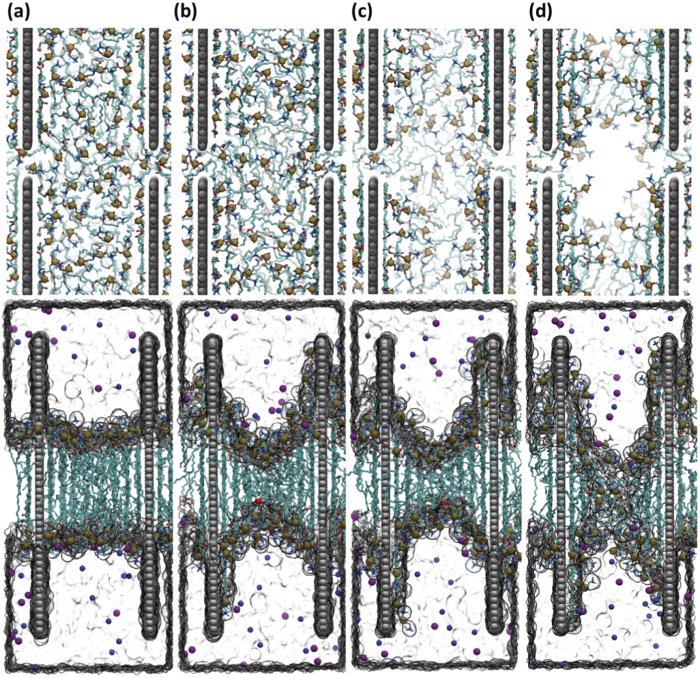
Top and side views of a representative simulation trajectory at (**a**) 1 ns, (**b**) 35 ns, (**c**) 70 ns and (**d**) 100 ns. As the simulation progresses, more and more lipids are drawn onto the graphene surfaces, leading to increased curvature in the inter-graphene membrane. After 100 ns, the membrane is perforated, and water flows through the newly formed pore. The plotting scheme here mirrors that in Fig. 5 (to clearly render the pore, we have hidden ions in the top panels).

Figure 6 shows top- and side-view snapshots of the graphene-membrane system taken from a representative trajectory. Strong dispersion interactions between graphene and phospholipid tails seem to drive the perforation process: lipid molecules are extracted from the membrane and drawn onto the graphene surface, resulting in increased negative curvature within the interstitial membrane segment. Lipid extraction begins immediately after the simulation starts; more and more phospholipids accumulate on exposed graphene surfaces as the inter-graphene membrane loses density (here, we define the inter-graphene membrane to exclude the lipid layer in direct contact with graphenes). At around 90 ns, the depleted membrane loses its integrity, and a pore forms in a location central to the graphene quartet. Consistent with our data of MD simulation, we also found that the size of light spots increased with the extension of incubation time in experiment. Eventually, deformed and dead cells resulting from membrane damage caused by lipid extraction (Movie S1) were detected.

**Figure 7:**
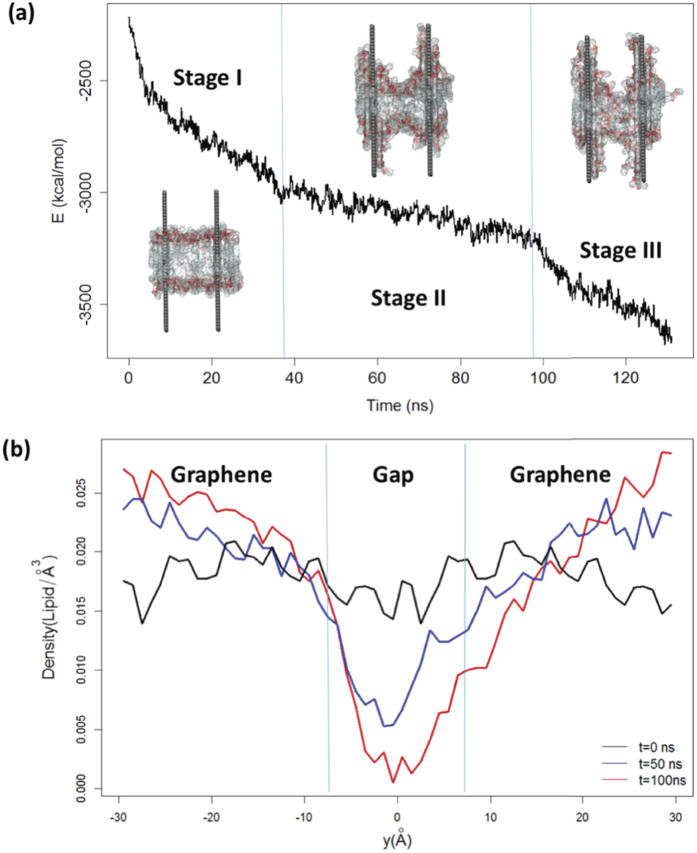
(**a**) vdW energy profile for interactions between lipids and graphenes. The trend can be roughly divided into three stages — a fast lipid extraction stage (Stage I); a metastable stage featuring balance between dispersion forces and membrane stress (Stage II); and a stage defined by perforation and further lipid extraction (Stage III). A representative snapshot from each stage is shown in the plot, with the lipid membrane rendered in a molecular surface representation. Different lipid occupancies on the nanosheet surfaces are characteristic of each stage. (**b**) Lipid density projected onto the y-axis (see Fig. 5c for an illustration of the projection axis) at different stages of pore formation. The density profile switches quickly from a homogeneous state to a deep valley, as lipids in the gap area are transported to the inter-graphene region. A pore finally develops in the gap region as the red curve approaches zero.

We computed the vdW interaction energies between the graphene sheets and phospholipids (Fig. 7a and Figure S12), a quantity proportional to the contact area between the two groups. These dispersion energies reveal further details of the observed pore formation mechanism. In particular, the perforation process can roughly be divided into three stages. During the first stage (Stage I), phospholipids are quickly extracted onto the graphene surfaces, resulting in a steep decline in the vdW energy. The process then slows down significantly as the membrane enters a metastable state. During this stage (Stage II), the internal membrane tension roughly balances the graphene-mediated dispersion force pulling the membrane apart, and the membrane curvature is maintained at a relatively constant degree (compare Fig. 6b and c). During the final stage (Stage III), thermal fluctuations cause the membrane tension to finally yield to dispersive pulling forces, resulting in further lipid extraction, pore formation, and another sharp decrease in the vdW energy. The other trajectories collected exhibit a similar three-stage perforation process (Figure S12).

To further quantify this perforation mechanism, we calculated interstitial lipid densities during the three dynamical stages and projected these densities onto a designated y-axis (see Fig. 5c for coordinate definitions). The intervals (−30, −7.5) and (7.5, 30) describe the y-coordinates of two halves of graphene nanosheets with their most proximate edges positioned at −7.5 Å and 7.5 Å, respectively. The interval (−7.5, 7.5) represents the gap between these two edges, which here belong to a graphene nanosheet and its closest periodic image. At the beginning of the simulation (Stage I), the lipid density is relatively homogeneous along this whole axis, as expected for an unperturbed membrane. By 50 ns, however, we can see that the density profile has developed a deep valley, with the highest degree of depletion found in the gap region (Stage II). The lipids in this gap region are pulled toward the graphenes by attraction to nanosheet-adjacent lipid molecules, further stimulating the extraction process. The space directly between the flat graphene faces actually sees a lipid density enhancement, a phenomenon driven by strong dispersion interactions with the planar nanosheet surfaces. Accessible surface area near the center of each graphene face allows lipid molecules to diffuse freely upward, resulting in high surface occupancy by extracted lipids. After the adsorption of many lipid molecules, much of the region contained between the graphene faces has a higher-than-initial lipid population, although the central portion of the membrane (with respect to the x-axis shown in Fig. 5c) is thinner than a standard phospholipid bilayer. As Stage II progresses, the lipid population in the gap region nears a critical value. At the beginning of the third stage, the connected network among lipids in the gap region dissipates and a pore forms in the membrane (see Supporting Movie, top view). Water molecules enter the expanding pore and, in some cases, pass through to the opposite side of the bilayer (Stage III).

It is interesting to note that the pores observed here appear close to the edges of the graphenes in the quartet, suggesting this interstitial region is reliably weakened by lipid extraction. This edge-centric perforation mechanism is consistent with the previous experimental finding that nanosheet edges are important mediators of the toxical effects of graphenes^28^. We would like to emphasize, however, that a single edge belonging to a graphene of this approximate size seems to be incapable of inducing pore formation in cell membranes, as supported by our previous lipid extraction simulations involving a single graphene sheet. The perforation observed here results from cooperative lipid extraction mediated by multiple graphene nanosheets. The nanosheet quartet in our simulations created a confined space within which lipids were simultaneously attracted to graphenes in several directions, leading to critically weakened spots in the membrane. The drastic depletion of phospholipid molecules within this confined area ultimately led to the formation of a complete, water-permeable membrane pore (Fig. 4).

The observation of cooperative membrane perforation in our simulation system, of course, does not preclude the emergence of alternative perforation mechanisms driven by larger GO sheets. For example, if a single GO nanosheet were sized on the order of the linear dimension of a cell, that nanosheet might be capable of extracting a significant percentage of the phospholipids that make up an entire cell membrane. Establishing whether such isolated interactions could indeed cause membrane perforation, or whether some fundamental physical limitation prohibits this type of pore formation, represents and interesting subject of future study.

## Conclusion

In this work, we used both experiments and simulations to demonstrate that pristine graphene and graphene oxide nanosheets can induce pore formation on cell membranes. Electron micrographs show clear images of these pores, and molecular dynamics simulations reveal a molecular mechanism for perforation dependent on cooperative lipid extraction driven by several graphene nanosheets. The observed pore formation mechanism can roughly be divided into three stages: fast lipid extraction onto multiple graphene surfaces (Stage I); a metastable balance between dispersion forces and membrane tension in a region confined between these nanosheets (Stage II); and ultimately, perforation (Stage III). Lipids in the proto-pore region are transported onto graphene surfaces and into the inter-graphene area, critically depleting the membrane’s density at characteristic interstitial sites. A pore develops when the confined lipid supply is finally exhausted.

To our knowledge, this cooperative, lipid-extraction-based pore formation process offers a unique perspective on the molecular mechanisms of graphene cytotoxicity. At low graphene concentrations, individual nanosheets should still be able to penetrate cell membranes and enter cell interiors. The damage caused by this graphene insertion (induced, for example, by a disruption of protein-protein interactions^28^), however, likely pales in comparison to the damage inflicted by perforation and a resultant influx of water into the cytosolic compartment. Discerning the graphene/GO concentrations at which excessive pore formation occurs likely represents an important step for ensuring the biosafety of graphene nanotechnologies. Below this limit of membrane perforation, graphene-based nanomaterials could be significantly more compatible with human systems of interest.

## Experimental Section

### Materials

GO was purchased from Chengdu Organic Chemical Company, Chinese Academy of Science. The A549 cell line was obtained from the Type Culture Collection of the Chinese Academy of Sciences, Shanghai, China. CCK-8 and Live/dead kits were purchased from Dojindo Molecular Technologies Inc. (Kumamoto, Japan) and Life Technologies Corporation (Carlsbad, CA, USA), respectively.

### Cell culture

A549 and Raw 264.7 cells were maintained in RPMI 1640 and DMEM media, respectively (Gibco, CA, USA), which were supplemented with 10% fetal bovine serum (FBS, Gibco, CA, USA), 1% penicillin-streptomycin solution, 1% L-glutamine and 1% nonessential amino acids and cultured at 37 °C and 5% CO_2_.

### Cytotoxicity detection

Cytotoxicity induced by GO was assessed by CCK-8 and live/dead assays. Briefly, A549 and Raw 264.7 cells were seeded on 96-well plates (Corning) at a density of 5,000 and 10000 cells per well, respectively, and cultured in complete culture medium containing 10% FBS. After 24 hours of incubation, A549 and Raw 264.7 cells reached ~80% confluence. Subsequently, for our CCK-8 experiments, A549 and Raw 264.7 cells were treated with GO in FBS-free medium at concentrations of 0, 1, 10, 50 and 200 μg/ml for 24 hours. Cells were then washed with PBS three times. CCK-8 was added to each well at a rate of 1:10 of the medium, and cells were subsequently incubated at 37 °C for another hour. Absorbance of the CCK-8 solutions was measured using a microplate reader at a 450 nm wavelength. A live/dead assay was also used to characterize the cytotoxicity of GO. Staining was performed following the protocol contained within the kit. Live (green) and dead (red) cells were observed via fluorescence microscopy.

### Cell morphological observations after GO treatment

To perform cellular morphology observations after GO treatment, cells were seeded into 6-well plates (1 × 10^5^/well). GO was dispersed in FBS-free medium at concentrations of 0, 10, 50 and 200 μg/ml and added to the 6-well plates. Cells were cultured at 37 °C for 2 hours and 6 hours, and cellular morphologies were imaged by microscopy. For live cell imaging, after 1 × 10^5^ cells were seeded in confocal dish for 24 h, 50 μg/ml GO nanosheets were added. After GO treatment for 2 h, cells were imaged every 10 min for 990 min by a live cell imaging system (Olympus cell^R system).

### Autophagy detection

A549 cells were seeded into a 6-well plate at a concentration of 2.5 × 10^5^/well. After 24 hours of incubation at 37 °C and 5% CO_2_, cells were treated with 50 μg/ml GO. 3-MA, an inhibitor of autophagy, was added 0.5 h before GO treatment at a 3 mM concentration. 6 hours later, cells were washed three times with PBS and then stained with MDC for 20 min. Fluorescence microscopy was used to detect autophagosomes.

### Scanning electron microscope (SEM) experiment

SEM was used to detect stress/damage on cellular surfaces. Cells were first seeded onto a 24-well plate coverslip with FBS serum medium (containing 10% FBS). Then, 24 hours later, after achieving ~80% confluence, cells were treated in serum free medium (0% FBS) with or without GO (200 μg/ml) for 6 hours. Cells were then prefixed in 2.5% glutaraldehyde overnight at 4 °C followed by a postfix in 2% (vol/vol) aqueous osmium tetroxide for 1 hour. Cells were rinsed with PBS 3 times and then dehydrated in 30%, 50%, 70%, 80%, 90%, 95% and 100% ethanol. After dryness reached a critical point, the coverslips were coated using gold sputter deposition and imaged via field-emission SEM.

### Molecular Dynamics Simulations

We carried out MD simulations for graphene nanosheets interacting with POPC (1-palmitoyl-2-oleoyl-sn-glycero-3-phosphocholine) lipids that are present in eukaryotic cell membranes. The CHARMM force field^56^ was used for simulating POPC lipids. At the beginning of each simulation, two parallel graphene nanosheets (4.7 nm × 10.8 nm) were inserted into the model membrane (containing 108 lipids) perpendicularly. On each side of a graphene sheet, about 32 nm^2^ of surface area is exposed to water. The force field used for graphene was adapted from our previous study^18^. The graphene-membrane complex was further solvated in a 0.1 M NaCl electrolyte containing 20 Na^+^, 20 Cl^−^, and 10840 water molecules. The TIP3P water^57^,^58^ model was used in concert with a standard force field for ions^59^. Following similar protocols used in our previous work^60^,^61^,^62^,^63^,^64^, we invoked the NPT (P = 1 bar and T = 300 K) ensemble to equilibrate the system (with fixed graphene sheets and fixed z-coordinates for lipid phosphorus atoms). In our production simulations (NVT ensemble), the graphene sheets were kept fixed, but lipids were allowed to move freely around the graphenes.

The NAMD2.9 65 software package was used to conduct all MD simulations. Langevin dynamics were applied to all water oxygen atoms to maintain the temperature of the simulated system; a smooth cutoff (10–12 Å) was used for calculating van der Waals interactions. Electrostatic interactions were calculated using the particle-mesh Ewald (PME) method (grid size ~1 Å). The integration time-step in all simulations was 1 fs.

## Additional Information

**How to cite this article**: Duan, G. *et al*. Graphene-Induced Pore Formation on Cell Membranes. *Sci. Rep.* **7**, 42767; doi: 10.1038/srep42767 (2017).

**Publisher's note:** Springer Nature remains neutral with regard to jurisdictional claims in published maps and institutional affiliations.

## Supplementary Material

Supplementary Figures

Supplementary movie 1

Supplementary movie 2

Supplementary movie 3
