# Supplementary material for: Graphene-Induced Pore Formation on Cell Membranes
Source: Sci Rep. 2017 Feb 20;7:42767. doi: 10.1038/srep42767 (PMC5317030; doi:10.1038/srep42767)
Supplement: Supplementary Figures [file srep42767-s1.pdf]

Supporting Information: Graphene-Induced Pore Formation on Cell Membranes

Guangxin Duan[1,‡], Yuanzhao Zhang[2,‡], Binquan Luan[2], Jeffrey K. Weber[2], Royce W. Zhou[3], Zaixing Yang[1], Lin Zhao[1], Jiaying Xu[1], Judong Luo[1], and Ruhong Zhou[2,3*]

[1] Institute of Quantitative Biology and Medicine, SRMP and RAD-X, Collaborative Innovation Center of Radiation Medicine of Jiangsu Higher Education Institutions, Soochow University, Suzhou 215123, China; [2] IBM Thomas J. Watson Research Center, Yorktown Heights, NY 10598, USA; [3] Department of Chemistry, Columbia University, New York, NY 10027, USA

ǂ These authors contribute equally

*Corresponding author, E-mail: ruhongz@us.ibm.com

Movie S1: Live cell imaging of A549 cells after GO treatment (from 120 to 1100 min).

Movie S2: Top view of tmembrane perforation between graphene nanosheets.

Movie S3: Side view the membrane perforation between graphene nanosheets.

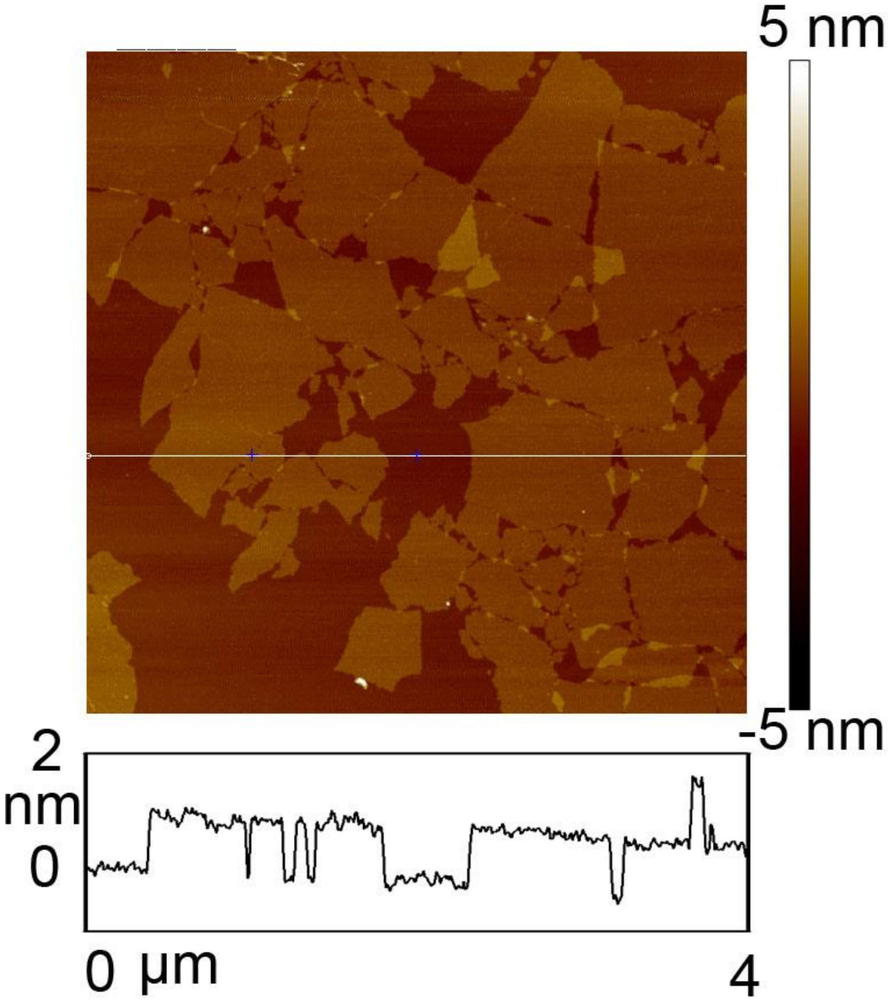

Figure S1: GO characterization by AFM. AFM images indicate a GO thickness of around 1 nm, which suggests a single-layer GO architecture consistent with previous results. The lateral sizes of the GOs ranged from 200nm to 700nm.

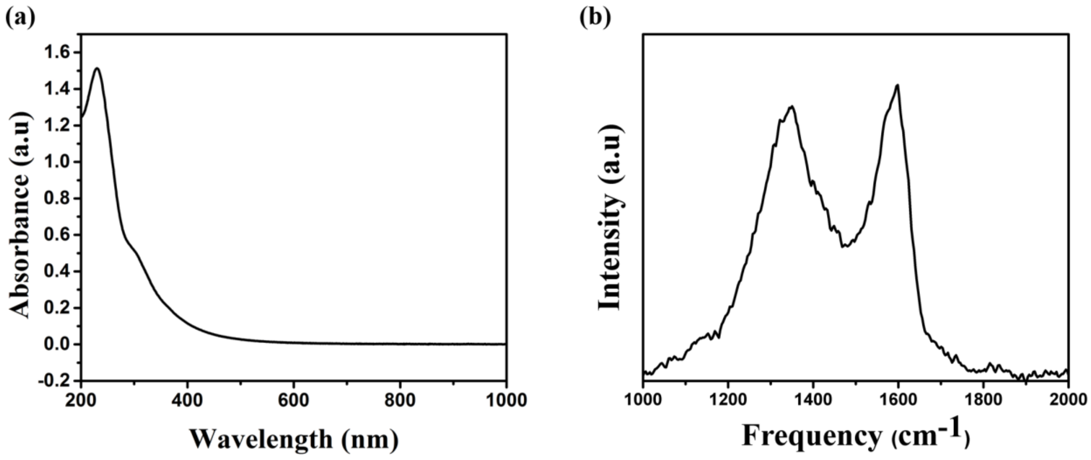

Figure S2. Characterizations of GO nanosheets by UV (a) and Raman (b) spectra. The main UV absorbance peak appeared at ~230nm, consistent with the location observed in previous studies. Raman spectra indicate that GO's characteristic D and G bands emerge, as expected, at ~1350 and 1598 cm^{-1}, respectively.

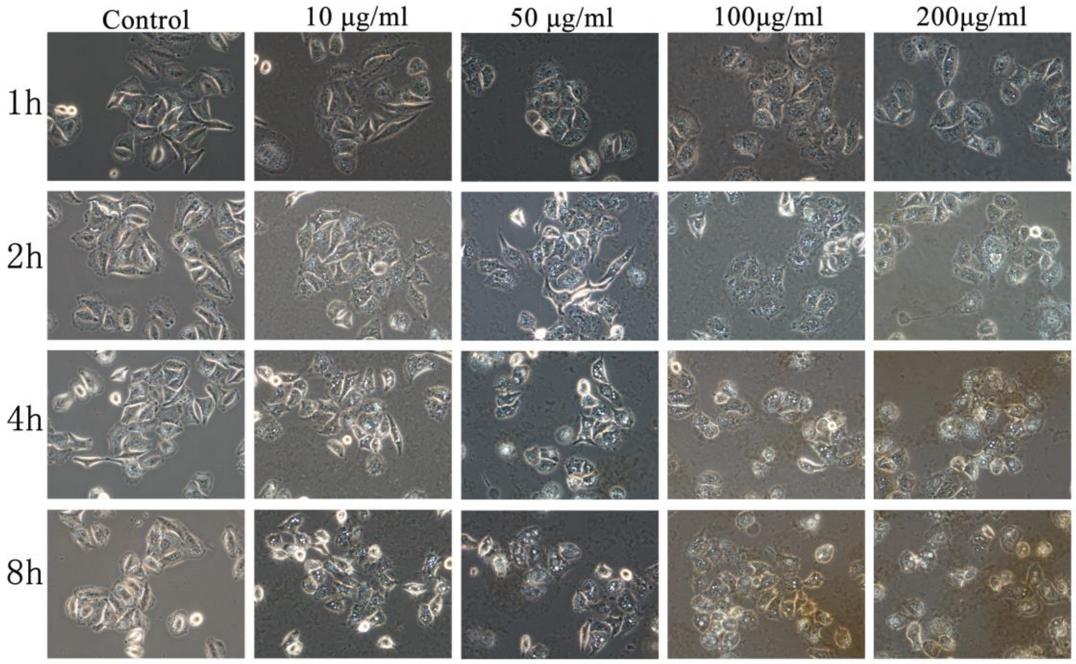

Figure S3. Light spots on A549 cells were detected after treated with 10, 50, 100 and 200 μg/ml GO for 1, 2, 4 and 8h. Light spots were observed within 1h for 10 μg/ml GO treatment and number and size increased with the increase of incubation time and treatment concentration. When the incubation time up to 4h and 8h and treatment concentration more than 100 μg/ml, however, number of light spots decrease significantly, which might result from shrinkage and death of cells.

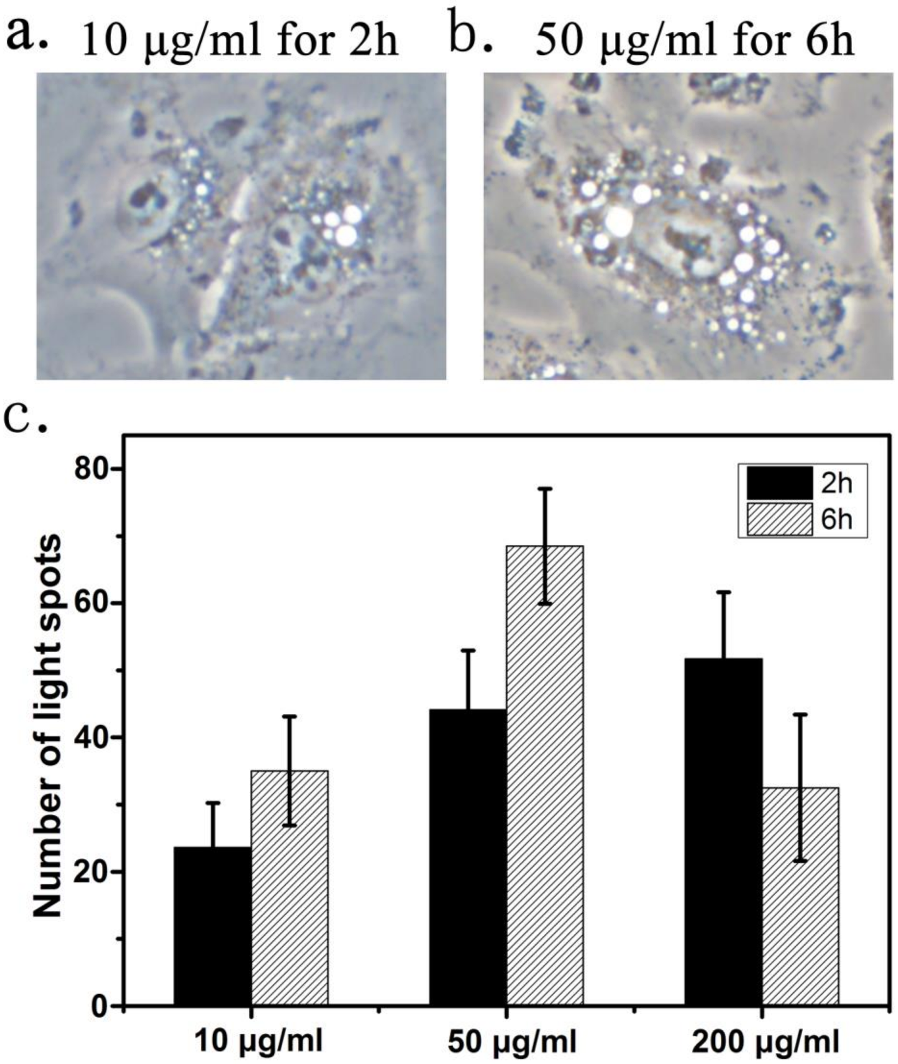

Figure S4. Light spots on A549 cells were counted after treated with GO nanosheets. About 23.6±6.6 light spots could be observed after 10 μg/ml GO treated for 2h.Up to approximate 68.5±8.6 holes could be detected on A549 under the condition of 50 μg/ml for 6h. The number of light spots was analyzed by counting. 10 cells were counted in each group and the number of light spots in each cells represent the mean±SD of light spots in 10 cells of each group.

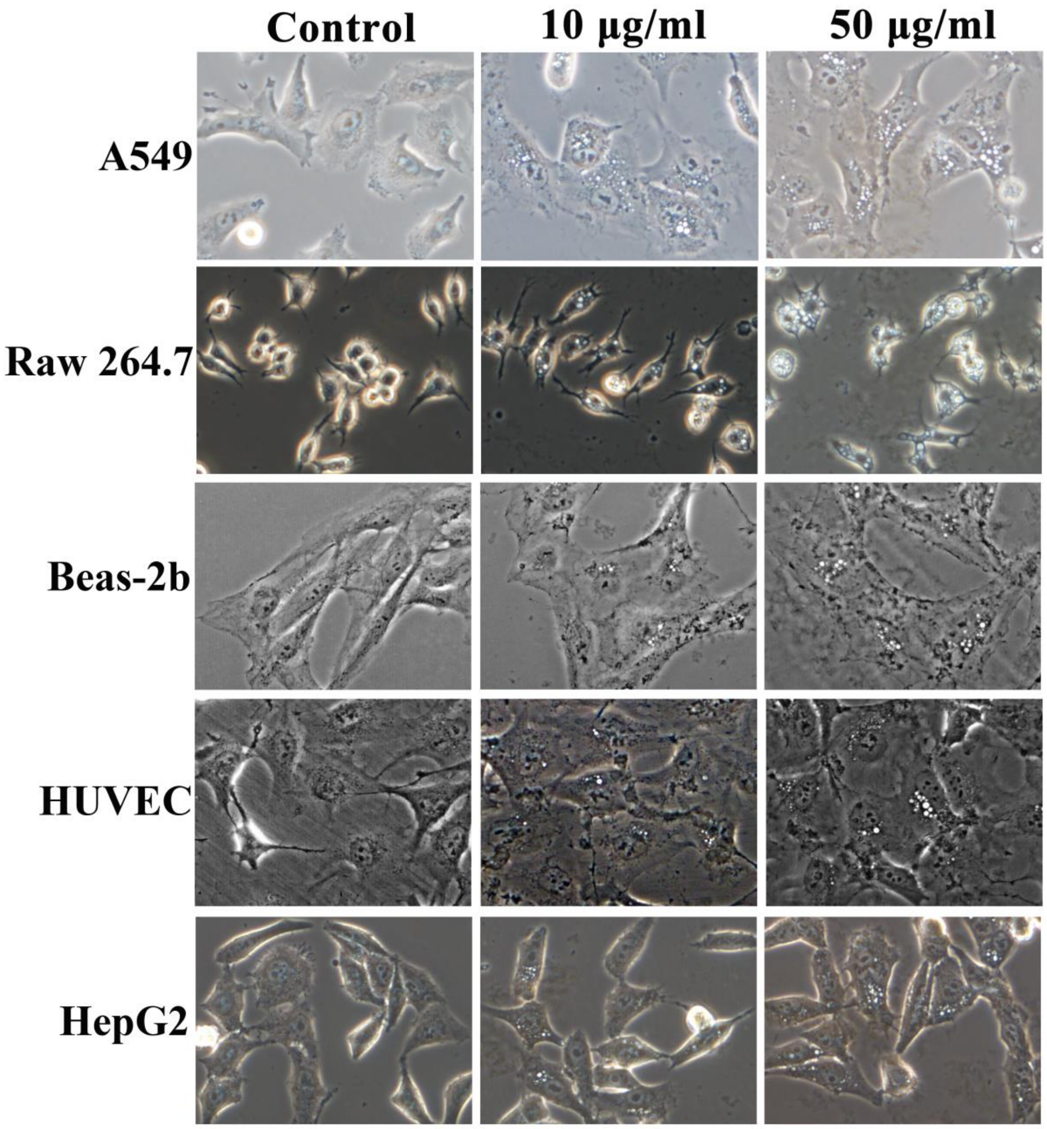

Figure S5. Appearance of GO-induced light spots in five cell lines. Light spots were found observed in Beas-2b, HUVEC and HepG2 cell lines, but were more prominent on A549 and Raw 264.7 cells.

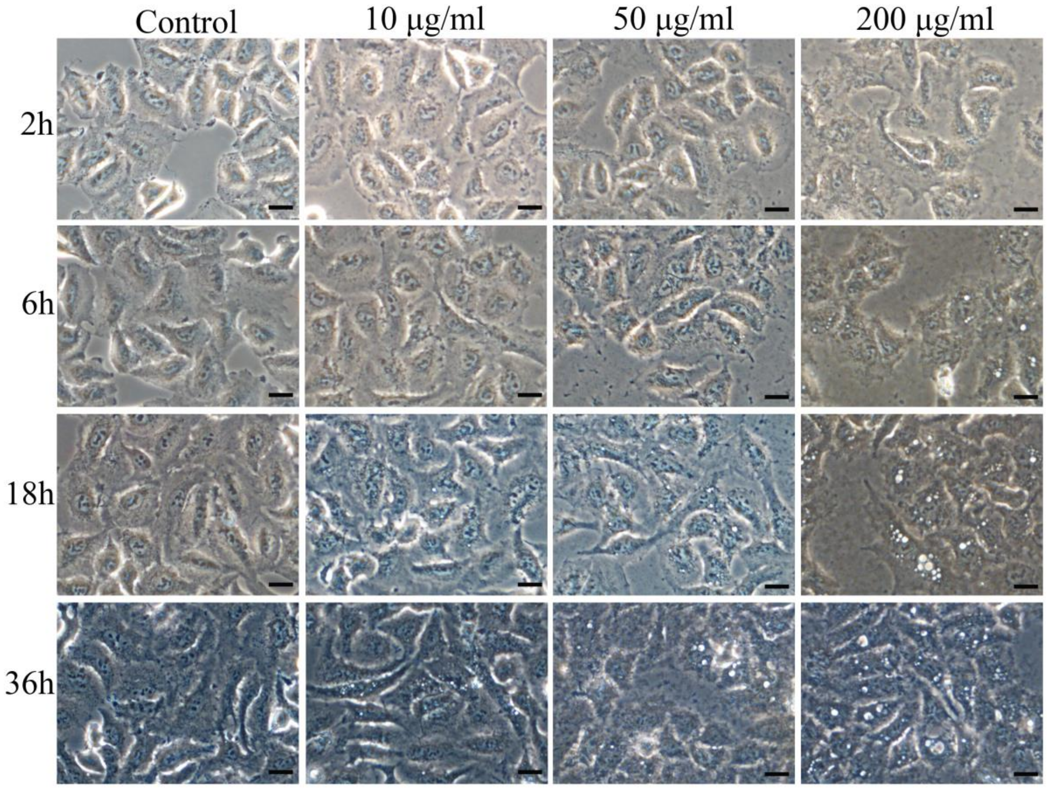

Figure S6： Morphologies of A549 cells as imaged by optical microscopy after GO treatment in complete serum medium containing 10% FBS. Scale bar=20 μm.

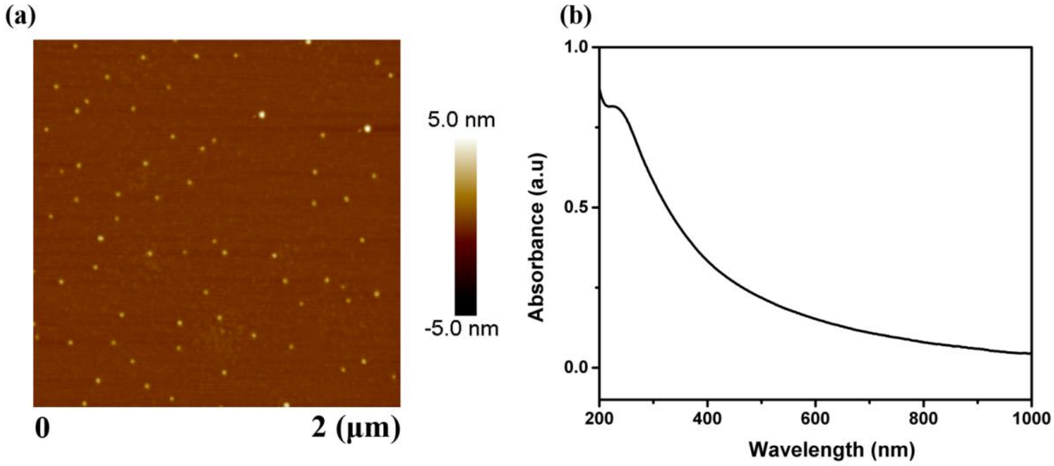

Figure S7. Characterization of GO-PEG by AFM (a) and UV spectroscopy (b). The average size of the PEG-GOs was about 50 nm, with a PEG layer height reaching 2-4 nm. The prominent UV absorbance peak moved from 230 nm in GO to ~222nm, a change that can be attributed to PEG conjugation.

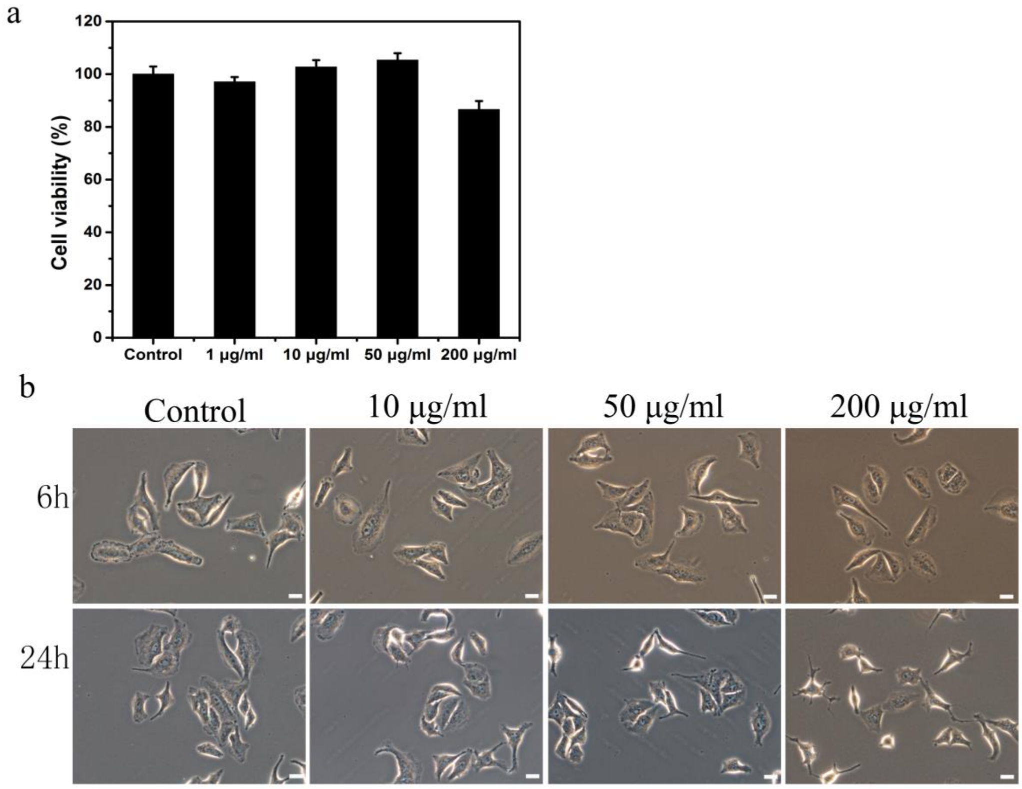

Figure S8: Observations of minimal PEGylated GO (PEG-GO) cytotoxicity. Cell viabilities (a) and morphologies (b) were analyzed with CCK-8 assays and optical microscopy after PEG-GO treatment. Scale bar=20 μm.

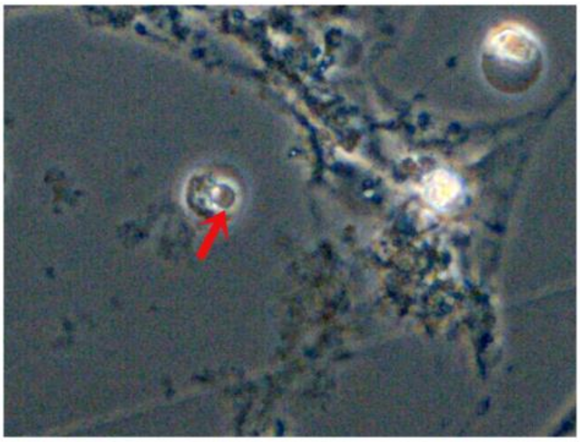

Figure S9. Pores (marked by arrows) found on the membranes of dead cells.

(a)

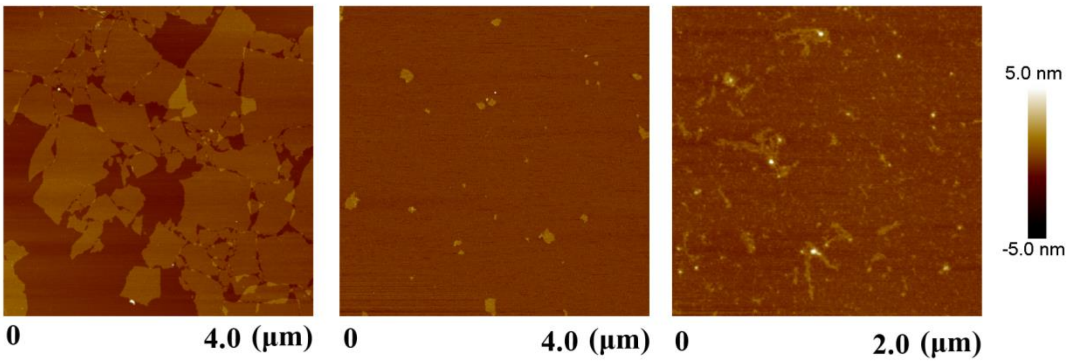

(b)

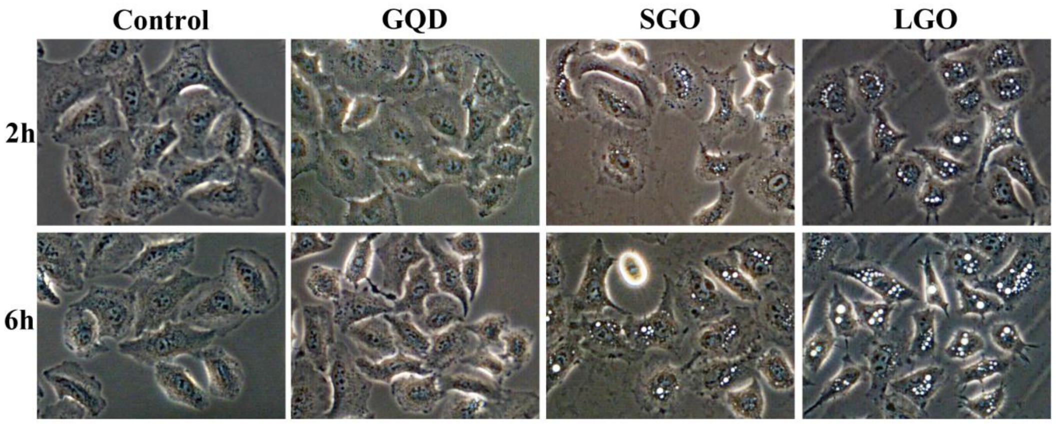

Figure S10. Increase in the number and extent of pores on A549 cells with increasing GO size. (a) Large-GO (LGO, 200-700nm, used in all the above experiments), small-GO (SGO, 50-150nm) and GO quantum dots (GQD, ~15nm), as characterized by AFM. (b) Pores induced by LGO, SGO and GQD. After exposure with 10 μg/mL GO for 2 and 6h, LGO induced the formation of the largest and most numerous pores, whereas the GQD produced the smallest and fewest perforations.

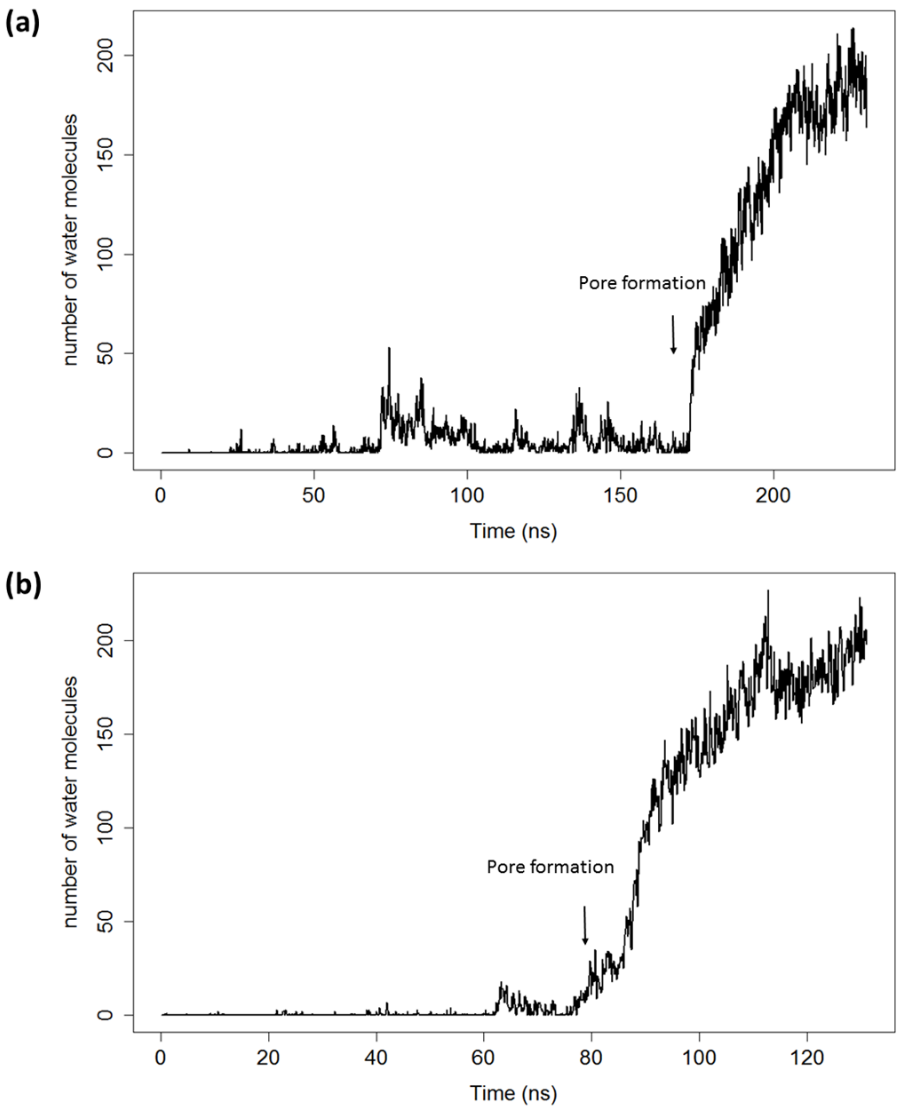

Figure S11: Number of water molecules inside the membrane as a function of time for two additional trajectories.

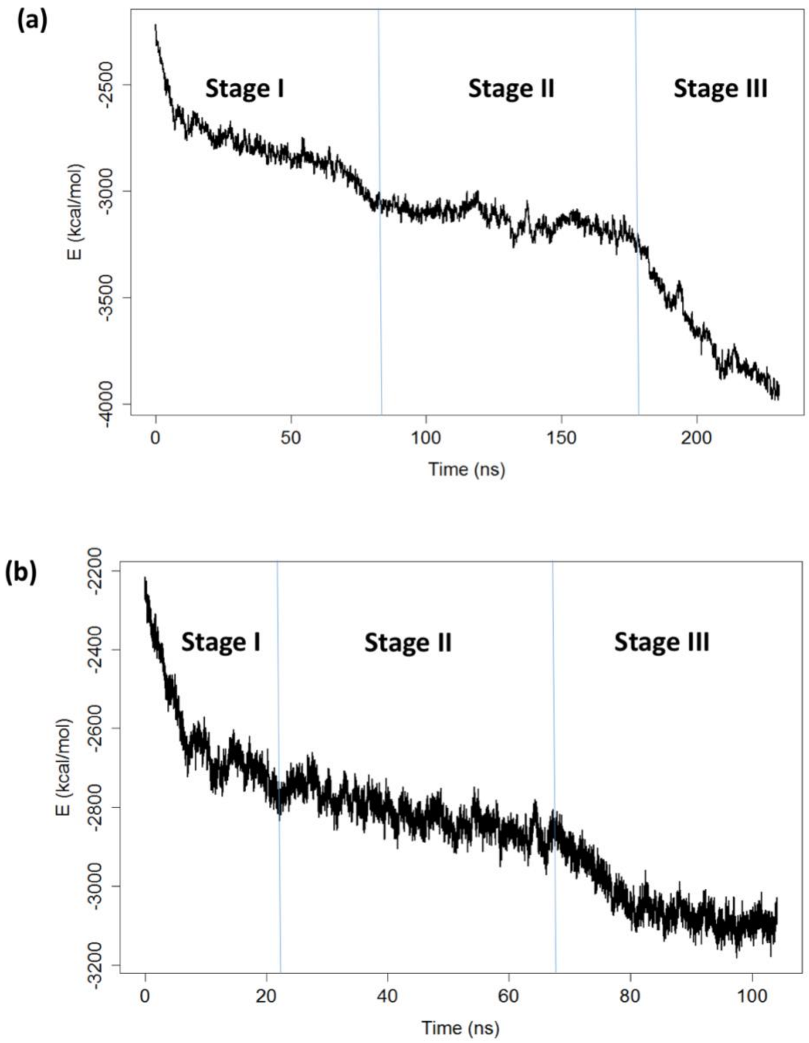

Figure S12: vdW energy profiles for graphene-membrane interactions corresponding to two additional trajectories.
